# Weather impact on airborne coronavirus survival

**DOI:** 10.1063/5.0024272

**Published:** 2020-09-01

**Authors:** Talib Dbouk, Dimitris Drikakis

**Affiliations:** University of Nicosia, Nicosia CY-2417, Cyprus

## Abstract

The contribution of this paper toward understanding of airborne coronavirus survival is twofold: We develop new theoretical correlations for the unsteady evaporation of coronavirus (CoV) contaminated saliva droplets. Furthermore, we implement the new correlations in a three-dimensional multiphase Eulerian–Lagrangian computational fluid dynamics solver to study the effects of weather conditions on airborne virus transmission. The new theory introduces a thermal history kernel and provides transient Nusselt (Nu) and Sherwood (Sh) numbers as a function of the Reynolds (Re), Prandtl (Pr), and Schmidt numbers (Sc). For the first time, these new correlations take into account the mixture properties due to the concentration of CoV particles in a saliva droplet. We show that the steady-state relationships induce significant errors and must not be applied in unsteady saliva droplet evaporation. The classical theory introduces substantial deviations in Nu and Sh values when increasing the Reynolds number defined at the droplet scale. The effects of relative humidity, temperature, and wind speed on the transport and viability of CoV in a cloud of airborne saliva droplets are also examined. The results reveal that a significant reduction of virus viability occurs when both high temperature and low relative humidity occur. The droplet cloud’s traveled distance and concentration remain significant at any temperature if the relative humidity is high, which is in contradiction with what was previously believed by many epidemiologists. The above could explain the increase in CoV cases in many crowded cities around the middle of July (e.g., Delhi), where both high temperature and high relative humidity values were recorded one month earlier (during June). Moreover, it creates a crucial alert for the possibility of a second wave of the pandemic in the coming autumn and winter seasons when low temperatures and high wind speeds will increase airborne virus survival and transmission.

## I. INTRODUCTION

Aerosol of respiratory droplet transmission is a primary vehicle for the rapid spread and continued circulation of viruses in humans.^1–5^ There is also evidence that environmental conditions can affect virus transmission.^6,7^

The virions, i.e., the infectious particle designed for the transmission of the nucleic acid genome among hosts or host cells, are expelled from humans through coughing, sneezing, talking, or normal breathing and are immersed in a respiratory fluid. A critical factor for the transmission of the airborne virions is the saliva liquid carrier-droplet evaporation. If we have a better understanding of the evaporation process and its relation to climate effects, we can more accurately predict the evolution of virus concentration in space and in time and determine its viability rate, i.e., potential of virus survival.

There are many papers dedicated to the investigation of various fluid dynamics and heat transfer aspects of droplet evaporation, e.g., see Refs. 8 and 9 and references therein. Despite the importance of airborne droplet transmission, research regarding heat and mass transfer around and within respiratory droplets containing virions is scarce. Vejerano *et al.*^10^ showed that the chemical microenvironment immediately surrounding virions in droplets and aerosols is likely to be a critical determinant of their stability.

Droplet evaporation, in general, has been studied for various applications,^8^ but the evaporation of saliva droplets containing virus particles is not understood. So far, the theory for heat and mass transfer was based on the Nusselt (Nu) and Sherwood (Sh) number correlations of Ranz and Marshall,^11,12^ which defined Nu and Sh numbers a function of the Reynolds, Prandtl, and Schmidt numbers. However, the Ranz and Marshall^11,12^ formulas concern a steady-state heat and mass transfer of flowing spherical particles made of a single material. Many authors incorrectly adopted them in the literature for different transient cases, neglecting the multi-material (mixture) properties of spherical droplets.

In this study, we develop new correlations for heat and mass transfer for droplet evaporation, which provide Nu and Sh as a function of time, Reynolds, Prandtl, and Schmidt numbers, as well as including fluid and thermodynamic properties of the virus. We have introduced the thermodynamic properties of virions as a liquid. The correlations include the liquid saliva portion inside the droplet through the molecular formula for the phospholipid of a coronavirus (CoV) capsid structure. Furthermore, we introduce transient effects and a thermal history kernel. The new correlations account for the virion concentration in saliva droplets and their effect on the unsteady evaporation process.

The knowledge of climate effects on SARS-CoV-2 and other virus survival and transmission is limited, as recent studies have shown.^13–18^ Virus infections are more common during winter times, and it has been mentioned that the CoV can be transmitted for a period of up to two weeks at low temperatures and low humidity.^19^ However, the mechanisms of climate parameters influencing the virus survival, concentration, and disease transmission remain unknown. There is an urgency to understand the climate parameters on COVID-19, particularly while facing the possibility of continued spread of the virus worldwide. Using the new heat and mass transfer correlations, we present a study of the effects of relative humidity (RH), environmental temperature, and wind speed on the respiratory cloud and virus viability. The results provide new insight into the influence of the above parameters, which contradicts current understanding.

## II. SURVIVAL OF AIRBORNE CONTAMINATED SALIVA DROPLETS

The coronavirus (CoV) COVID-19 has been consistently detected in the saliva of infected persons.^20^ Recent studies confirmed that human saliva constitutes high potential for the diagnostic and transmission of COVID-19 among humans.^21–23^

**FIG. 1.**
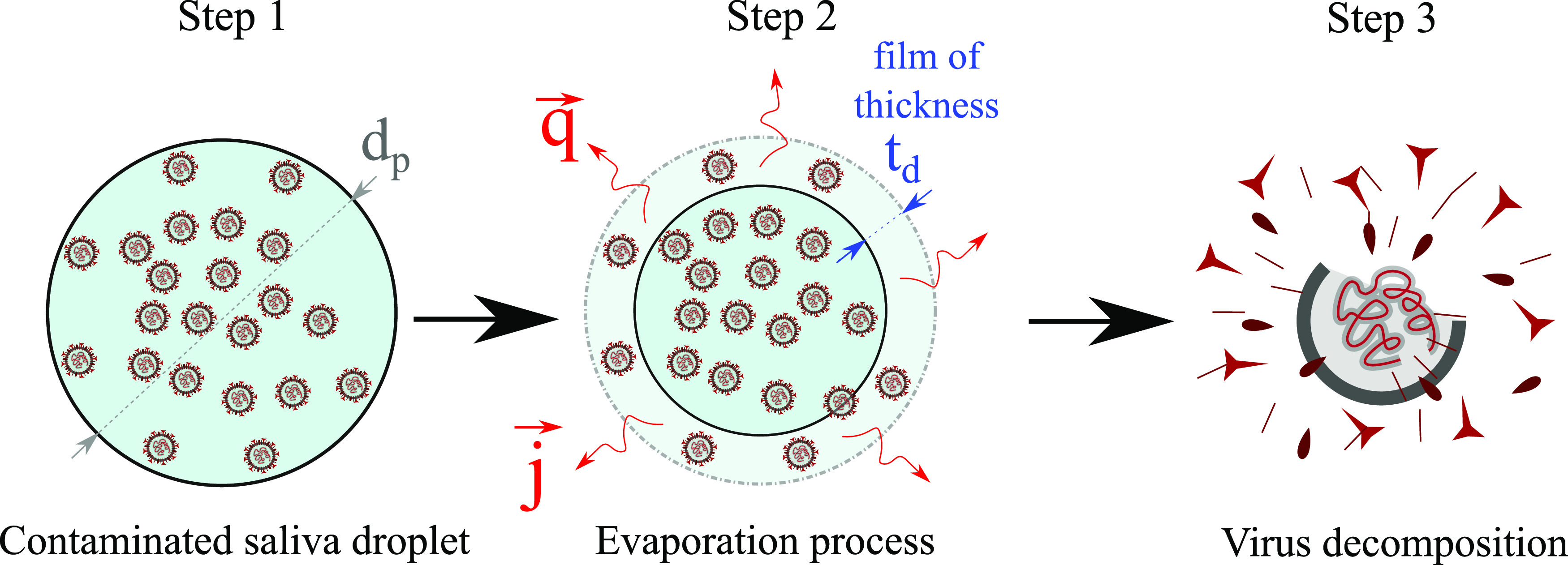
A three-step mechanism of virus destruction due to evaporation. Step 1: A saliva droplet of diameter *d*_*p*_ is initially contaminated by CoV particles of *C*_0_ concentration. Step 2: The evaporation process begins with **q** and **j** being the surface heat and mass fluxes due to saliva droplet advection in air and evaporation. *t*_*d*_ denotes the thickness of the evaporating vapor film. Step 3: Virus decomposition and inactivation.

The mechanism of reduction of virus concentration due to evaporation can be described in three steps (Fig. 1): (a) we assume a contaminated saliva droplet. (b) The process includes the saliva droplet advection and evaporation. An evaporating vapor film of thickness *t*_*d*_ forms around the droplet. (c) The complete droplet evaporation leads to the virus structure decomposition and inactivation. Therefore, evaporation is crucial for limiting airborne virus transmission.

### A. Importance of climate conditions

The influence of relative humidity and temperature on the airborne virus survival is a complex topic. The outcomes of past experimental studies are contradictory, and the mechanism of the effect of relative humidity and temperature on virus survival remains mostly unknown. Previous studies from the literature reported that relative humidity is a significant climate determinant in the transmission of influenza.^24–26^ Past research also attempted to optimize and control the humidity level to reduce the virus survival in an indoor environment.^27^

Yang and Marr^28^ reviewed different conjectures regarding the relationship between relative humidity and the virus in aerosols. Water activity, surface inactivation, and salt toxicity could play an essential role in the virus’s persistence in experimental studies.

Sobsey and Meschke^29^ suggested in a WHO report that enveloped viruses containing a lipid membrane have higher survivability at lower RH. In contrast, non-enveloped ones tend to be more stable at higher RH. However, the above hypothesis does not explain several other phenomena, e.g., the Rous sarcoma virus (RSV) and infectious bovine rhinotracheitis virus (IBRV), which are enveloped and appear more stable at higher RH, or the pigeon pox virus, which is insensitive to RH; see Ref. 28 and references therein.

Harper^30^ studied the RH effects on vaccinia, influenza, Venezuelan equine encephalomyelitis, and poliomyelitis experimentally. At each RH level, he found that viable survival of airborne viruses was better at lower temperature values than at higher ones. He illustrated that poliomyelitis virus has the best durability at high RH, while all the other three viruses have the best survivability at low RH. After 50 years, many researchers still believe and emphasize that the coronavirus is less likely to survive at high RH.^19^ They used this message to propose solutions for reducing COVID-19 by increasing the RH in indoor environments. Unfortunately, there exists a vital enigma behind this topic. The findings by Webb *et al.*^31^ presented another contradictory theory, which was recently confirmed to be true.^28^ Yang and Marr^28^ showed that the effect of RH on airborne virus survival is profoundly affected by the initial composition of the spraying medium used to create the experimental airborne droplet. When the spraying medium is water, the airborne viruses survive better at high RH.^31^ The above is due to the added water through spraying that delays evaporation and not to the environmental humidity that exists in the surrounding air; hence, the conclusions of past research were misleading. By developing a new theory and modeling of heat and mass transfer, we have performed 3D computational fluid dynamics (CFD) simulations showing how the behavior of the virus changes across a range of RH and temperatures. We will show that airborne viruses can survive at high RH, which is in agreement with the experimental findings of Webb *et al.*^31^ and the review of Yang and Marr.^28^

### B. Heat and mass transfer of an evaporating virus-contaminated saliva droplet

**FIG. 2.**
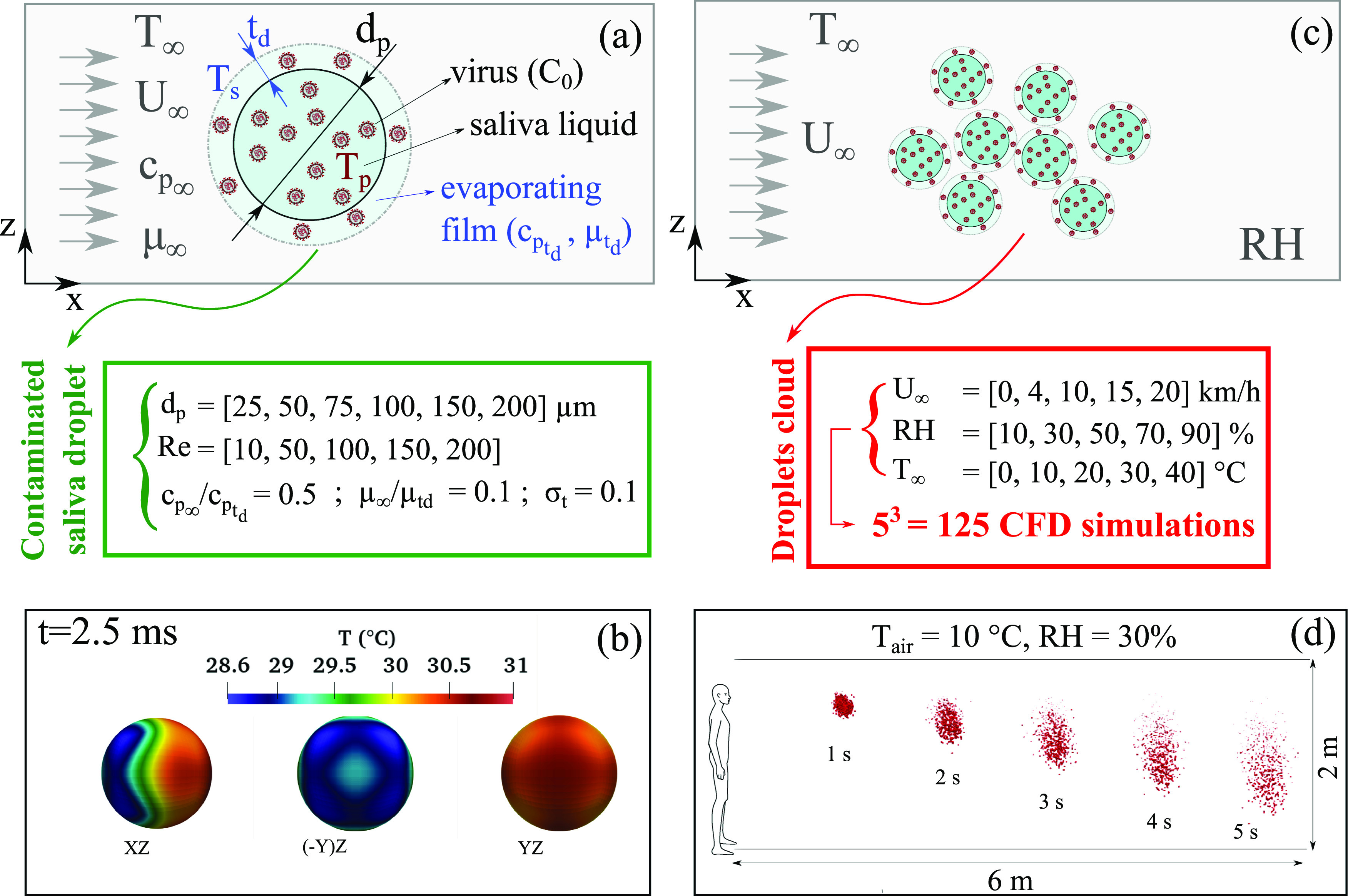
A schematic representation for quantifying the effect of weather on the heat and mass transfer rate of a CoV-contaminated saliva droplet. [(a) and (c)] Schematics of the computational domain showing the weather conditions (wind speed *U*_*wind*_ = *U*_*∞*_, environment temperature *T* = *T*_*∞*_, the relative humidity RH, and the initial concentration *C*_0_ of CoV in saliva). *t*_*d*_ denotes the thickness of the evaporating vapor film. (b) An example at Re = 200 [see Eq. (1)] that shows the temperature distribution at *t* = 2.5 ms around a contaminated saliva droplet from different perspective plane views. (d) An example at *U*_*wind*_ = 4 km/h showing the transport and evaporation of a contaminated cloud of saliva droplets between 1 and 5 s. The green and red boxes show the parameters used to conduct the different 3D CFD simulations for the freestream of air flowing around a contaminated saliva droplet (green box: single sphere droplet; red box: droplet cloud). The cloud is expelled at a cough speed of *U*_*cough*_ = 8.5 m/s, as explained in the work of Dbouk and Drikakis.^32^

When a contaminated saliva droplet is expelled from the mouth or nose into the surroundings at speed *U*_*cough*_, it exchanges heat and mass with ambient air (evaporation). We consider unsteady free-stream airflow at speed *U*_*wind*_ = *U*_*∞*_ around a spherical saliva droplet at different Reynolds numbers, as illustrated in Fig. 2(a). The saliva droplet contains CoV particles at an initial concentration of *C*_0_. We take into account the conjugated heat transfer in the airflow and inside the saliva droplet. Figure 2(b) shows an example at Re = 200 [Eq. (1)] for the solution of the temperature distribution at *t* = 2.5 ms around a contaminated saliva droplet. We also consider hundreds of CFD simulations for a contaminated cloud of saliva droplets expelled at a *U*_*cough*_ = 8.5 m/s [see Fig. 2(c)] to quantify the influence of the weather conditions on the airborne CoV viability, thus virus transmission. Figure 2(d) shows an example at *U*_*wind*_ = 4 km/h, RH = 10%, and *T*_*∞*_ = 10 °C for the cloud transport and evaporation between 1 s and 5 s.

The convection inside the saliva droplet was neglected due to the low temperature gradients and high viscosity ratio (*ρ*_*p*_/*ρ*_*f*_ ≫ 1). The transient compressible Navier–Stokes and energy equations for the airflow and the heat diffusion equation for the saliva droplet are solved iteratively over a three-dimensional refined computational grid. Mesh sensitivity analysis was conducted and the size of the grid was decided according to a grid convergence index proposed by Ref. 33 applied in our case to the averaged heat flux *q* (see Fig. 1) computed at the surface of the saliva droplet. Second-order schemes in both space and time in the framework of a finite volume method^34^ discretization were applied.

Since the capsid of CoV represents the largest surface area of the virus particle, its thermal and physical properties must be taken into account. The CoV capsid is a phospholipid bilayer that has properties very similar to dipalmitoylphosphatidylcholine (DPPC). DPPC has an effective density close to water.^35^ Its heat capacity is 2167 J kg^−1^ K,^36^ and its thermal conductivity is about 0.48 W m^−1^ K^−1^.^37^

We performed several CFD simulations to quantify the heat transfer for an airflow around a CoV-contaminated saliva droplet at different Reynolds and Prandtl numbers,

$$Re_{\infty }=\frac{\rho_{\infty }U_{\infty }d_{p}}{\mu_{\infty }},$$ (1)

$$Pr_{\infty }=\frac{{c_{p}}_{\infty }\mu_{\infty }}{k_{\infty }},$$ (2)

where *μ*_*∞*_ is the dynamic viscosity, *k*_*∞*_ is the thermal conductivity, ${c_{p}}_{\infty }$ is the heat capacity, and *ρ*_*∞*_ is the density. The subscript *∞* symbol denotes airflow properties far away from the spherical droplet (see Fig. 2). The new correlations emerging from the present study are presented in this section together with a historical overview of the evolution of the Nusselt number correlation.

To quantify the heat and mass transfer, we conducted hundreds of different 3D CFD simulations for the freestream of air flowing around a contaminated saliva droplet. Figure 2The green and red boxes in Fig. 2 show the several parameters used to conduct the numerous CFD simulations.

The averaged local Nusselt number $\hat{Nu}$ describing the ratio of convective to conductive heat transfer is given by

$$\hat{Nu}\;=\;\frac{ĥ\;d_{p}}{k_{\infty }},$$ (3)

where *k*_*∞*_ is the initial thermal conductivity of air at ambient temperature and pressure. $\hat{h}$ is the averaged convective heat transfer coefficient defined at the droplet surface,

$$\hat{h}\;=\;\frac{\hat{q}}{\left(T_{S}-T_{\infty }\right)}$$ (4)

and

$$\hat{T_{S}}\;=\;\frac{\int_{S}T\;dS}{\pi {d_{p}}^{2}},$$ (5)

where *S* is the spherical surface boundary of the saliva droplet and $\hat{q}$ is the averaged local heat flux per unit area (W/m^2^) computed at the interface between the saliva droplet and the surrounding airflow. $\hat{T_{S}}$ is the averaged temperature of the saliva droplet surface, and *T*_*∞*_ is the airflow free-stream temperature (*T*_*∞*_ = 293 K).

Experimental studies of heat and mass transfer for a single spherical droplet are scarce because of the difficulties in accomplishing accurate experiments, especially when small microdroplets are involved at varying Reynolds and Prandtl numbers. The classical theory widely used today for estimating the evaporation rate of spherical droplets in many applications originates from Ranz and Marshall^11,12^ who correlated the Nusselt number to the Reynolds and Prandtl numbers,

$$Nu=2\;+\;0.55\;Re^{1/2}\;Pr^{1/3}.$$ (6)

Ranz’s and Marshall’s studies^11,12^ were complementary to the results by Fuchs,^38^ Wells,^39^ and Frossling^40^ back in the 1930s. Following Ranz and Marshall,^11,12^ several authors researched to improve and extend the Nusselt number correlation as a function of the Reynolds number; see the work of Acrivos^41^ and Brenner.^42^ The Ranz and Marshall^11,12^ correlation was enhanced after several years by Refs. 43 and 44. The result of the above efforts was the following equation:

$$Nu=2\;+\;\left(0.4\;{Re}_{\infty }^{1/2}+0.06\;{Re}_{\infty }^{2/3}\right)\cdot {Pr_{\infty }}^{0.4}{\left(\mu_{\infty }/\mu_{t_{d}}\right)}^{1/4}.$$ (7)

For spheres immersed in infinite media, Whitaker^44^ showed that in the laminar boundary layer region, the contribution to the Nusselt number should be of the form *Re*^1/2^*Pr*^1/3^, while in the wake region, Richardson^43^ proposed the form *Re*^1/2^*Pr*^2/3^. The above lead to Eq. (7) with the exponents’ values obtained by fitting with the experimental data of Kramers^45^ and Vliet and Leppert.^46^

Feng and Michaelides^47–50^ highlighted and quantified the effects of high and low Peclet numbers and the influence of arbitrary shapes and viscous particles on the Nusselt number correlation. Moreover, they shed light on the unsteady effect of heat transfer studying a sphere at small Peclet numbers. They studied the unsteady heat conduction equation from a small sphere considering terms that are analogous to those found in the equation of motion of a sphere (“Basset terms”) as it was shown recently by Duan *et al.*^51^ They derived their results from asymptotic analysis and showed that the transient Nusselt number is of the form $Nu=2\left(1+1/\sqrt{\pi t^{*}}\right)\;+\;O\left(Pe^{1+}\right)$, with t^*^ = *O*(1). To our knowledge, there are no experimental measurements for the unsteady evaporation process of liquid droplets immersed in infinite medium that could be used in validating the above term. However, the asymptotic analysis is accurate and derived from first principles. Thus, we have adopted the above term in the present modeling of contaminated liquid saliva droplets.

Yearling and Gould^52^ improved the Nusselt vs Reynolds number correlation to account for the influence of the relative turbulence intensity (*σ*_*t*_) of an upstream airflow on the evaporation rate of liquid droplets. They introduced a correction term of the form $\left(1+\sigma_{t}^{0.843}\right)$. Lee, Hsu, and Pfender^53^ investigated the role of the heat capacity ratio between the far-field airflow and the zone near the surface of the droplet where evaporation takes place—the above is relevant to the *t*_*d*_ layer in Fig. 1—and they introduced a multiplication term of the form ${\left({c_{p}}_{\infty }/{c_{p}}_{t_{d}}\right)}^{0.38}$.

Based upon the above findings, in the present study, we propose a new correlation for the transient Nusselt number that accounts for the transient effects, turbulent airflow intensity, and for the influence of the different properties of the evaporating mixture layer (*t*_*d*_ layer in Fig. 1). This new correlation for the unsteady Nusselt number is under the following form:

$$\begin{array}{ccc} Nu\left(t\right) & = & 2\left(1+1/\sqrt{\pi t^{*}}\right)\;+\;\left(0.4\;{Re}_{\infty }^{1/2}+0.06\;{Re}_{\infty }^{2/3}\right)\\ & & \times \;{Pr_{\infty }}^{0.4}{\left(\mu_{\infty }/\mu_{t_{d}}\right)}^{1/4}\left(1+\sigma_{t}^{0.843}\right){\left({c_{p}}_{\infty }/{c_{p}}_{t_{d}}\right)}^{0.38}. \end{array}$$ (8)

As explained above, all the terms in Eq. (8) can be found in the correlations addressed by Ref. 44, 47, 52, and 53. Equation (8) will be used later to describe the heat transfer of evaporating airborne contaminated saliva droplets, as well as mass transfer, thanks to the analogy by Chilton and Colburn,^54^ accurately. This new transient correlation in Eq. (8) (henceforth labeled as “new theory”) has been validated (see Sec. II C) by comparison to the steady-state correlations of Ranz and Marshall,^11,12^ Richardson,^43^ and Whitaker,^44^ which have been derived from experimental measurements.

The subscript *t*_*d*_ denotes the property at the vapor film of thickness *t*_*d*_ that depends on the initial concentration of CoV in saliva droplets (see step 2 in Fig. 1). The time t^*^ is made dimensionless by using the diffusion timescale *τ*_*c*_ as

$$t^{*}=t\;/\;\tau_{c}\text{ with }\tau_{c}=\frac{{d_{p}}^{2}\rho_{d}{c_{p}}_{d}}{4k_{\infty }},$$ (9)

where *d*_*p*_ is the liquid saliva droplet diameter, *ρ*_*d*_ is the droplet initial density, and ${c_{p}}_{d}$ is the initial heat capacity.

Due to the low temperature values of the surrounding air flow (T < 40 °C) and following Prandlt–Blasius–Pohlhausen,^55^ one can assume that the mass transfer between the air flow and the contaminated saliva droplet surface occurs within a spherical diffusion film of thickness *t*_*d*_ (Fig. 1),

$$t_{d}\;=\;\frac{d_{p}}{0.6\;Sc^{1/3}\;{Re}_{\infty }^{1/2}}.$$ (10)

According to Chilton and Colburn,^54^ an analogy exists between heat and mass transfer correlations. Therefore, the Nusselt, Nu, and Prandtl, Pr, numbers in Eq. (8) can be replaced by the Sherwood and Schmidt numbers, respectively,

$$\begin{array}{ccc} Sh\left(t\right) & = & 2\left(1+1/\sqrt{\pi t^{*}}\right)\;+\;\left(0.4\;{Re}_{\infty }^{1/2}+0.06\;{Re}_{\infty }^{2/3}\right)\\ & & \times \;{Sc_{\infty }}^{0.4}{\left(\mu_{\infty }/\mu_{t_{d}}\right)}^{1/4}\left(1+\sigma_{t}^{0.843}\right){\left({c_{p}}_{\infty }/{c_{p}}_{t_{d}}\right)}^{0.38}. \end{array}$$ (11)

The Sherwood number describes the ratio of convective mass transfer to the diffusive mass transport,

$$Sh\;=\;\frac{h_{m}}{D/d_{p}},$$ (12)

where D is the mass diffusion coefficient of the evaporating film of thickness *t*_*d*_ diffused into the airflow and *h*_*m*_ is the film convective mass transfer coefficient.

The Schmidt number describes the ratio of momentum diffusion to mass diffusion,

$$Sc_{\infty }\;=\;\frac{\mu_{\infty }}{\rho_{\infty }\;D}.$$ (13)

The reduction in the droplet mass *m*_*p*_, i.e., a reduction in the droplet diameter *d*_*p*_, is described by the conservation equation

$$\frac{dm_{p}}{dt}=-\frac{Sh\left(t\right)}{3Sc_{\infty }}\frac{m_{p}}{\tau_{p}}\xi_{M},$$ (14)

where $\tau_{p}=\rho_{p}d_{p}^{2}/\left(18\;\mu \right)$ and *ξ*_*M*_ are the respective saliva droplet relaxation time and the dimensionless potential function driving the evaporation,

$$\xi_{M}\;=\;\frac{\left(\rho_{S}\;-\;\rho_{\infty }\right)}{\rho_{p}\left(C\right)},$$ (15)

where *ρ*_*∞*_ is the density far away from the droplet and *ρ*_*p*_(*C*) is the effective density of the liquid saliva droplet containing a concentration *C* of CoV particles such that *ρ*_*p*_ = *ρ*_*saliva*_ · (1 − *C*) + *ρ*_*CoV*_ · *C* with *C* = *C*_0_ at t = 0.

The mass fraction *Y* of the vapor film is defined as

$$Y_{S}\;=\;\frac{\rho_{S}}{\rho_{\infty }};\;\rho_{\infty }=\rho_{air},$$ (16)

where *ρ*_*S*_ is the density of the vapor at the droplet surface,

$$\rho_{S}\;=\;\frac{pM}{R\hat{T_{S}}},$$ (17)

where M is the molar mass of air, R its universal gas constant, and $\hat{T_{S}}$ is the Eulerian–Lagrangian averaged temperature at the saliva droplet surface,

$$\hat{T_{S}}\;=\;\left(2T_{\infty }+\hat{T_{p}}\right)/3,$$ (18)

with

$$\hat{T_{p}}\;=\;\frac{\int_{V_{p}}T_{p}\;dV_{p}}{V_{p}},$$ (19)

where *T*_*p*_ is the local temperature inside the saliva droplet computed at each computational cell and *V*_*p*_ is the volume of the saliva droplet. All of the above are time-dependent. The symbol ${}^{\wedge }$ denotes the averaging operation through a local integration process.

### C. Comparison of old and new theories for the Nusselt number

**FIG. 3.**
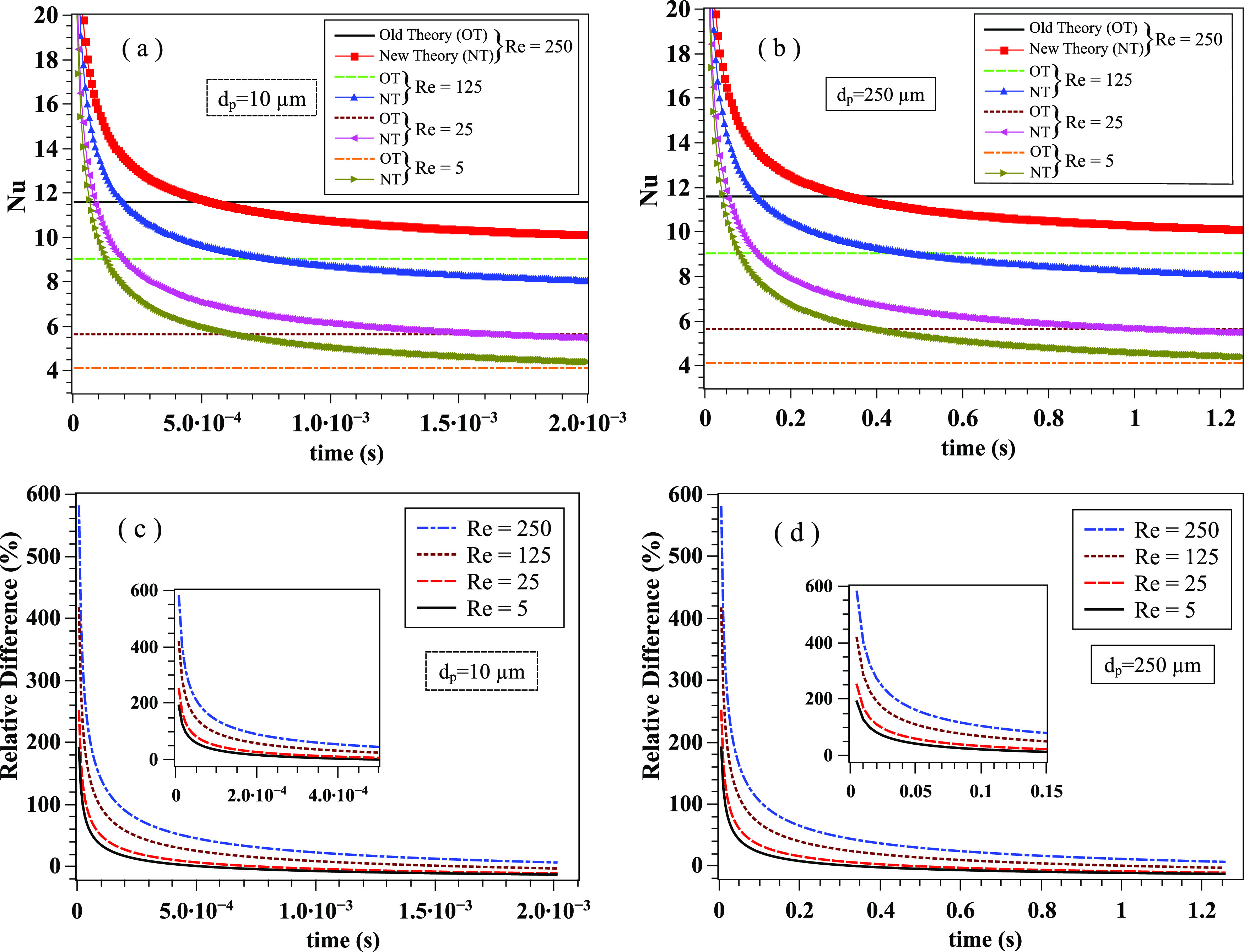
The transient Nusselt correlation [Eq. (8)] is compared with the steady-state Ranz–Marshall^11,12^ correlation [Eq. (6)] at different Reynolds numbers and droplet diameters. (a) Small saliva droplet of diameter *d*_*p*_ = 10 *µ*m; (b) larger saliva droplet of diameter *d*_*p*_ = 250 *µ*m; (c) relative difference with respect to the new theory for *d*_*p*_ = 10 *µ*m; and (d): relative difference with respect to the new theory for *d*_*p*_ = 250 *µ*m. The transient correlation results have been obtained for *Pr*_*∞*_ = 0.71, *σ*_*t*_ = 0.1, ${c_{p}}_{\infty }/{c_{p}}_{t_{d}}=0.5$, $\mu_{\infty }/\mu_{t_{d}}=0.9$, *ρ*_*d*_ = 1000 kg · m^−3^, ${c_{p}}_{d}=4180\;J\cdot kg^{-1}\cdot K^{-1}$, and *k*_*∞*_ = 0.026 W · m^−1^ · K^−1^.

**FIG. 4.**
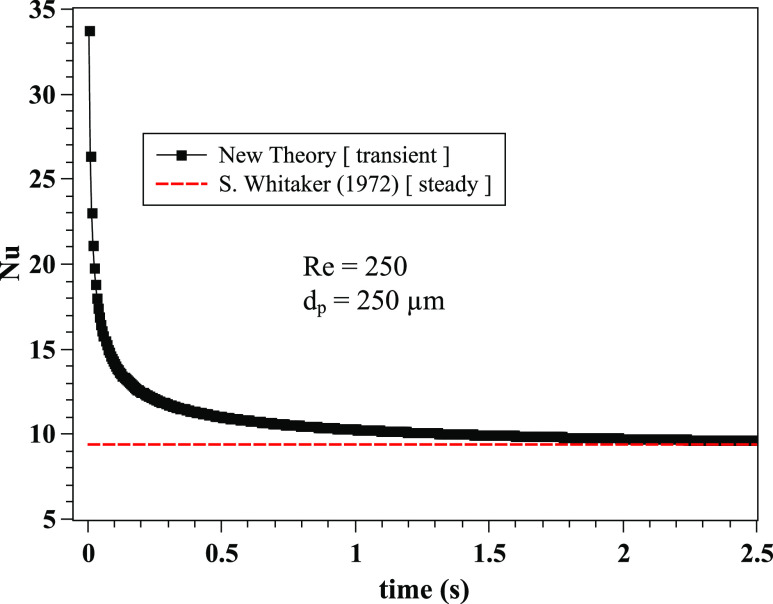
The transient Nusselt correlation [Eq. (8)] is compared with the steady-state Whitaker^44^ correlation [Eq. (7)]. The results concern the case of Re = 250 and *d*_*p*_ = 250 *µ*m. The transient correlation results have been obtained for *Pr*_*∞*_ = 0.71, *σ*_*t*_ = 0.1, ${c_{p}}_{\infty }/{c_{p}}_{t_{d}}=0.5$, $\mu_{\infty }/\mu_{t_{d}}=0.9$, *ρ*_*d*_ = 1000 kg · m^−3^, ${c_{p}}_{d}=4180\;J\cdot kg^{-1}\cdot K^{-1}$, and *k*_*∞*_ = 0.026 W · m^−1^ · K^−1^.

The importance of an unsteady correlation [Eq. (8)] compared to the widely used steady-state correlation of Ranz–Marshall^11,12^ [Eq. (6)] is illustrated in Fig. 3. We show that the new theory (labeled NT) has an important effect on the Nusselt number (similarly on the Sherwood number) at larger saliva droplets. For the droplet diameter *d*_*p*_ is about 250 *µ*m and *t* < 0.2 s, the old theory (labeled OT) underestimates the Nusselt number with the relative difference ranging between 10% and 580%. As time passes by, the difference is reduced, e.g., for *d*_*p*_ = 250 *µ*m and *t* > 0.2 s, the relative difference is between 1% and 10% [Fig. 3(d)]. For small droplet diameters, e.g., *d*_*p*_ = 10 *µ*m [Figs. 3(a) and 3(c)], the NT still has an important effect on the Nusselt number at early times, *t* ≤ 5 · 10^−4^ s. Furthermore, we compare the new transient Nu correlation with the steady-state Nu correlation^44^ [Eq. (7)] for Re = 250 and *d*_*p*_ = 250 *µ*m (Fig. 4). The new correlation converges to similar values to Whitaker’s correlation for the steady-state case. As expected, there are important deviations at early time instants. We note two things: (a) any differences between the new and old correlations in the asymptotic limit (steady-state) are not a matter of an error. The old correlations emerged from experimental fitting and are valid only for a range of the Reynolds numbers. For example, the Ranz and Marshall correlation^11,12^ is known to behave well at low Reynolds numbers values, while Whitaker’s^44^ is known to act better at higher Reynolds numbers. (b) Unsteady heat transfer experiments on evaporation of contaminated saliva droplets do not exist due to the short time and length scales. The results from Figs. 3 and 4 reveal that transient effects must be taken into account in the Nu correlation for accurate prediction of heat and, by analogy, mass transfer by similarity.

## III. RESULTS

We have employed the Eulerian–Lagrangian fully-coupled CFD model of Dbouk and Drikakis^32^ and implemented the new theoretical transient correlations [Eqs. (8) and (11)] that describe the heat and mass transfer at the microscopic scale of a contaminated saliva droplet. The three-dimensional computational domain comprises a conical injector (30°) of saliva droplets applied at the height of 1.7 m mimicking a mouth print. We have chosen a conical injector instead of a detailed mouth print as in the previous studies^32,56^ because the study focuses on the effect of the environmental parameters on saliva droplet cloud dynamics far away from the mouth. The precise form of mouth print plays an essential role only near the mouth.

The boundary conditions are the same as in the work of Dbouk and Drikakis^32^ subject to two modifications. Both the wind speed and the droplets injection start at time *t* = 0. The ground temperature is equal to the ambient air. The period of injection is 0.12 s representing a mild cough. Using the CFD models mentioned above in conjunction with the new theoretical models, we present below the results and analysis of the effects of relative humidity, environmental temperature, and wind on the airborne droplet transmission.

The approach we have followed in investigating the effects of weather conditions follows a similar path as in Dbouk an Drikakis,^32^ i.e., we investigate the cloud dynamic and strength of the airborne droplet cloud away from the subject.

**FIG. 5.**
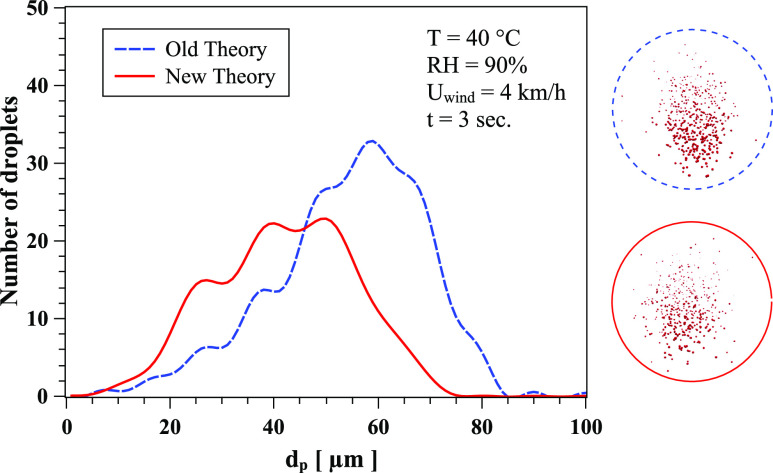
The effect of Nusselt and Sherwood correlations on the evaporation and local distribution of saliva droplets at t = 3 s, RH = 90%, T = 40 °C, and *U*_*wind*_ = 4 km/h. The circles show the corresponding form of the cloud dispersion obtained from CFD simulations. Dashed line circle: old theory; solid line circle: new theory.

**FIG. 6.**
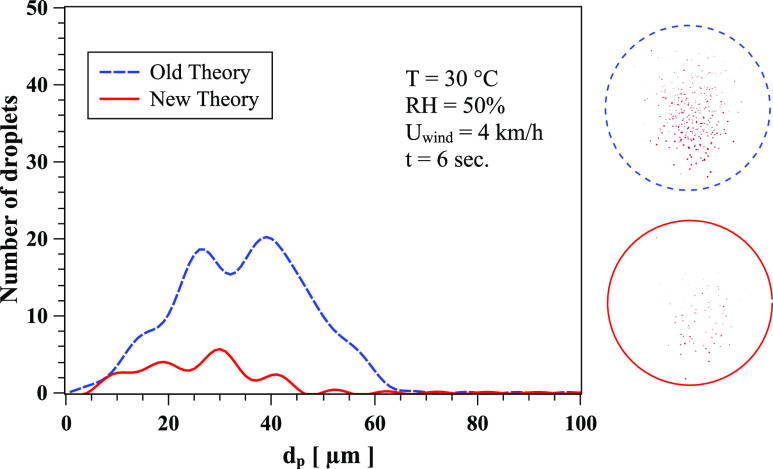
The effect of Nusselt and Sherwood correlations on the evaporation and local distribution of saliva droplets at t = 6 s, RH = 50%, T = 30 °C, and *U*_*wind*_ = 4 km/h. The circles show the corresponding form of the cloud dispersion obtained from CFD simulations. Dashed line circle: old theory; full line circle: new theory.

Figure 5 shows the quantitative effect of the new theory (correlation) on the evaporation rate. At t = 3 s, RH = 90%, T = 40 °C, and *U*_*wind*_ = 4 km/h, the total number of droplets is overpredicted by the old theory.^11,12^ Similar behavior occurs at t = 6 s, RH = 50%, T = 30 °C, and *U*_*wind*_ = 4 km/h (Fig. 6). The droplet spectrum is a result of both the new correlation and the contributions from the dynamics of the cloud and droplet/droplet interactions. The crossing of the curves in Fig. 5 is a result of the complex dynamics of the droplet cloud in addition to the evaporation process.

**FIG. 7.**
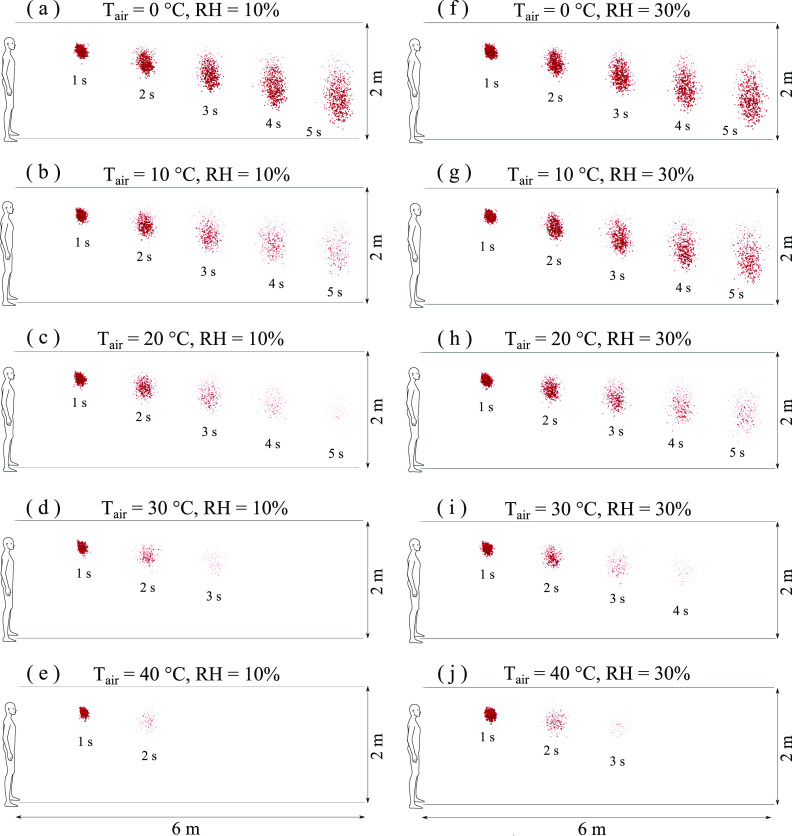
Weather impact on virus transmission. Influence of air temperature: [(a)–(e)] RH = 10%; [(f)–(j)] RH = 30%; wind speed: 4 km/h. Wind direction is from left to right.

**FIG. 8.**
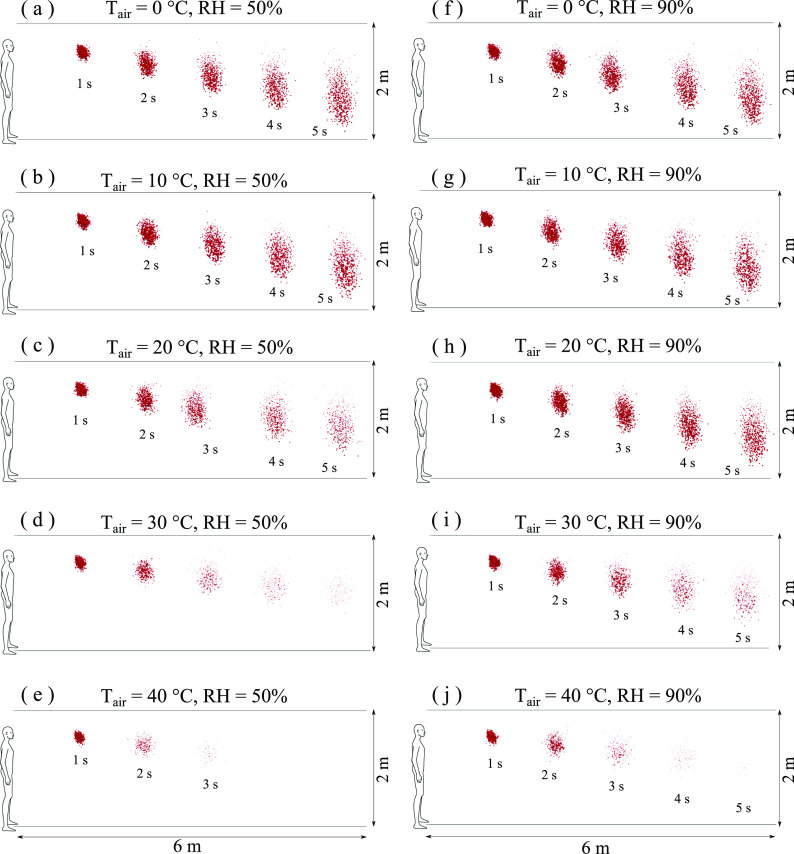
Weather impact on virus transmission. Influence of air temperature: [(a)–(e)] RH = 50%; [(f)–(j)] RH = 90%; wind speed: 4 km/h. Wind direction is from left to right.

Figures 7 and 8 show the results for a wind speed of 4 km/h and different temperatures and RH. At low RH, 10%–30%, with moderate to low temperatures (*T* ≤ 20 °C), the contaminated droplet cloud travels a distance of 6 m in 5 s. When the temperature increases to 30 °C and 40 °C, the droplets evaporate faster and the cloud travels a shorter distance. For example, at T = 20 °C and t = 5 s, comparing Figs. 7(c)–7(h), the increase in RH from 10% to 30% results in an essential reduction of the total number of contaminated saliva droplets. Furthermore, at the same RH = 30%, comparing Figs. 7(g)–7(i), the increase in temperature from 10 °C to 30 °C results at t = 5 s in total evaporation of the contaminated saliva droplets.

Figure 8 shows that when the RH increases from lower (10% and 30%) to higher (50% and 90%) values, the droplets become more resistant to evaporation. At high temperature (30°) and high RH (90%) [Fig. 8(i)], a significant rise in the total number of contaminated saliva droplets occurs, thus increasing the airborne virus viability. At higher temperatures and RH, the droplet cloud shrinks from an elongated form to a spherical one followed by higher dispersion; see Figs. 8(h) and 8(i) at t = 5 s. The above effects could explain the late pandemic acceleration observed in many crowded cities around the middle of July 2020 (e.g., Delhi), where both high temperature and high relative humidity values were recorded one month earlier (during June).^57^ The findings should be taken into consideration regarding the possibility of a second pandemic wave in autumn and winter seasons where low temperatures and high wind speeds will increase airborne virus survival and transmission.

**FIG. 9.**
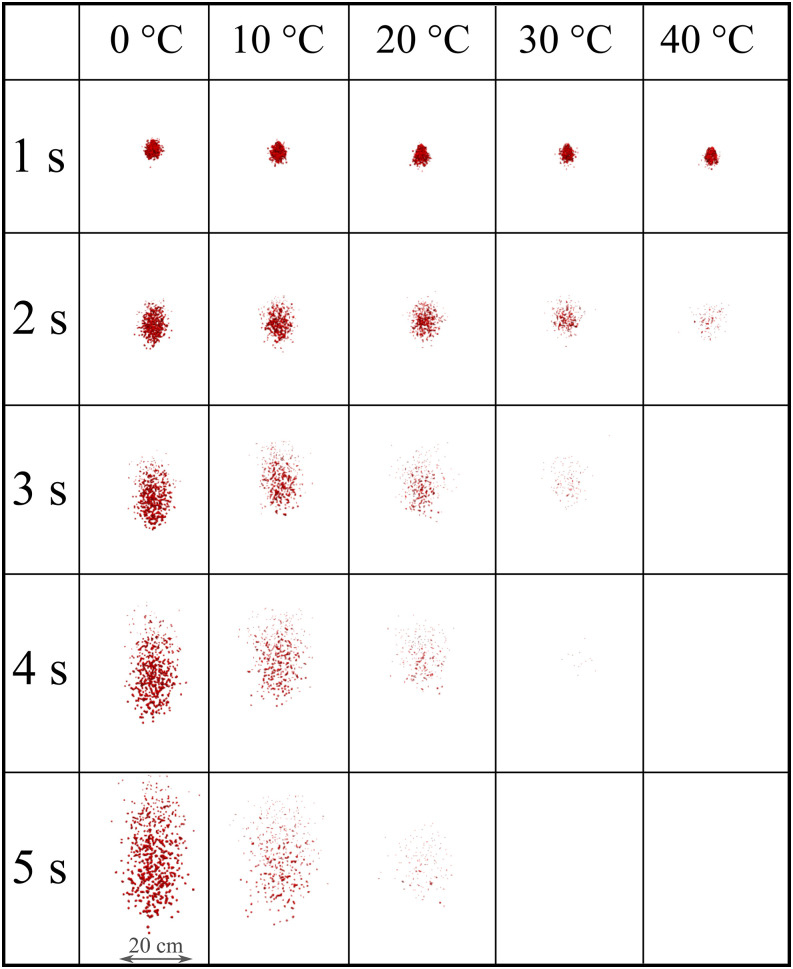
Weather impact on the transport and evaporation of airborne contaminated respiratory droplets at *U*_*wind*_ = 4 km/h and RH = 10%. Plane view from an observer located 8 m away from the source in the direction of the wind.

We present the strength of the droplet cloud with respect to time in Fig. 9 for RH = 10%. The cloud represents a spanwise view in the eyes of an observer situated 8 m away from the source. At RH 10%, the virus viability, which is linked to cloud disappearance, significantly decreases with the increase in temperature, mainly after 1 s of droplet transmission. For low to medium temperature values (0° to 20°), the cloud disperses as a function of time where vertical elongation occurs, resulting in an elliptic shaped cloud. At high-temperature values (30° to 40°), the cloud disperses more rapidly retaining a spherical-like shape due to a higher evaporation rate that results in the significant cloud evaporation around t = 3 s.

**FIG. 10.**
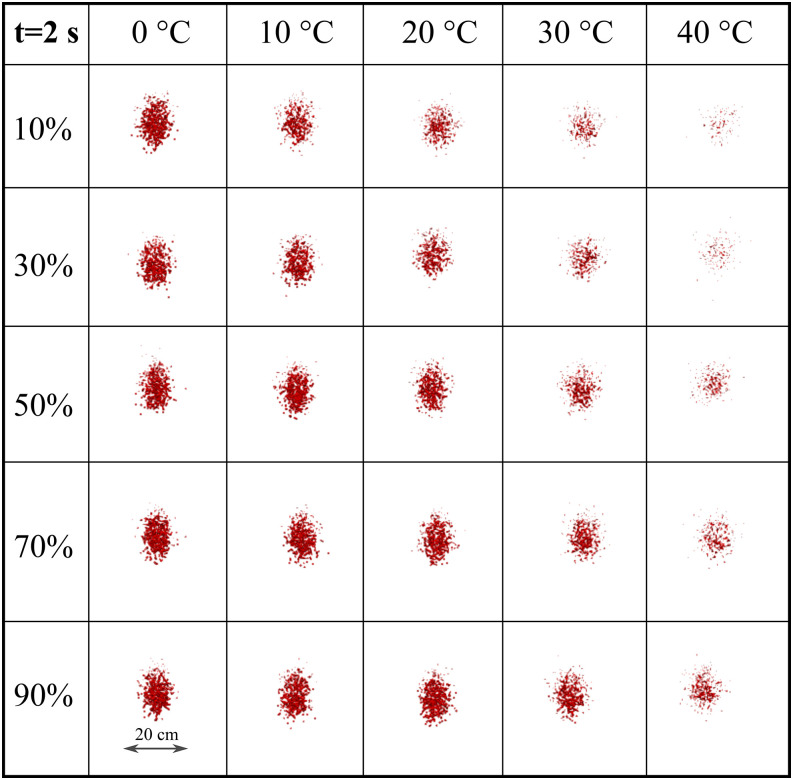
Weather effects (temperature and relative humidity) on the transport and evaporation of airborne contaminated respiratory droplets at *U*_*wind*_ = 4 km/h. Plane view at t = 2 s from an observer located 8 m away from the source in the wind direction.

**FIG. 11.**
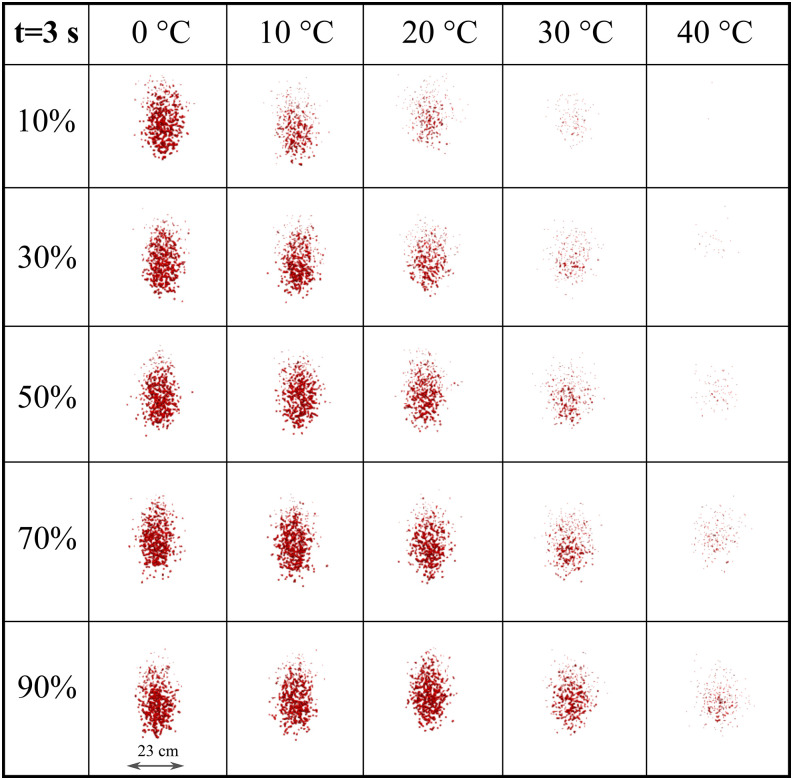
Weather effects (temperature and relative humidity) on the transport and evaporation of airborne contaminated respiratory droplets at *U*_*wind*_ = 4 km/h. Plane view at t = 3 s from an observer located 8 m away from the source in the wind direction.

To more precisely quantify the weather impact across a range of RH and temperatures, we present the cloud formation at 2 s (Fig. 10) and 3 s (Fig. 11). This matrix-like figure sheds light on the combined effect of RH and temperature leading to evaporation. For both simulated times and *U*_*wind*_ = 4 km/h, the results reveal that the virus viability is reduced at low RH and higher temperatures. Comparing Figs. 10 and 11, we see that at low to medium temperature values (0° to 20°), the cloud disperses more in the vertical direction. The evaporation is reduced, thus larger droplets remain in the environment and settle more rapidly due to gravity. At higher temperature (30° to 40°), low to medium RH (10%–50%) induces a higher evaporation rate, thus a more rapid decrease in the virus viability (Fig. 11).

**FIG. 12.**
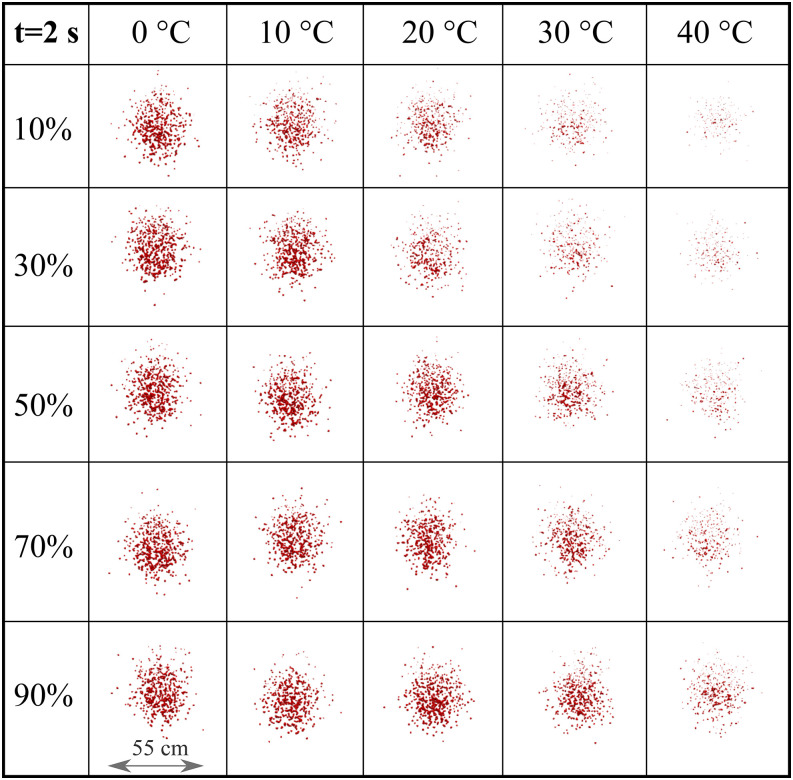
Weather effects (temperature and relative humidity) on the transport and evaporation of airborne contaminated respiratory droplets at *U*_*wind*_ = 10 km/h. Plane view at t = 2 s from an observer located 8 m away from the source in the direction of the wind.

**FIG. 13.**
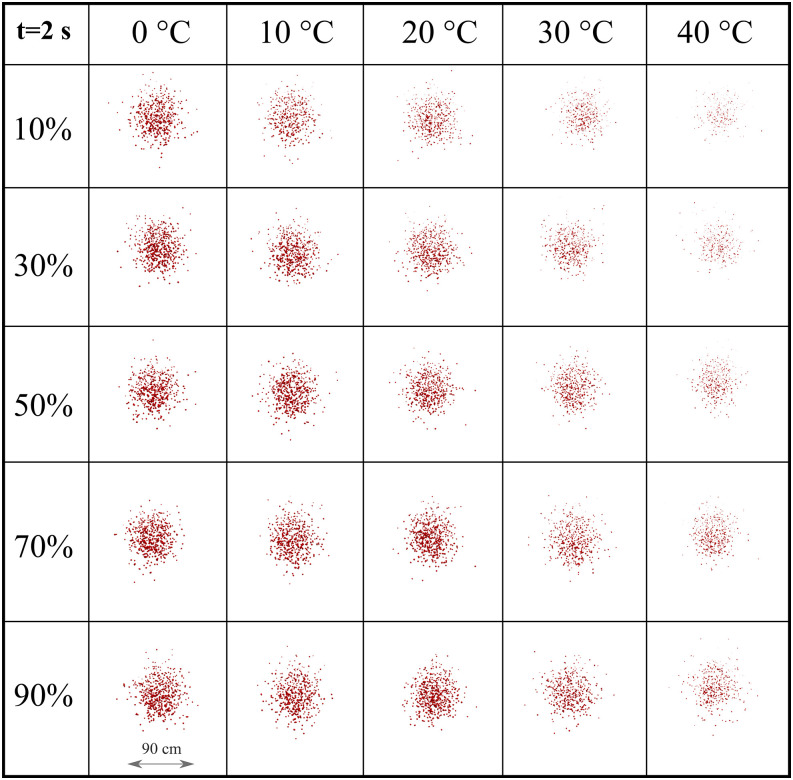
Weather effects (temperature and relative humidity) on the transport and evaporation of airborne contaminated respiratory droplets at *U*_*wind*_ = 15 km/h. Plane view at t = 2 s from an observer located 8 m away from the source in the direction of the wind.

We have also examined the effect of wind speed (*U*_*wind*_ = 10 km/h, and *U*_*wind*_ = 15 km/h) in combination with different RH and temperatures (Figs. 12 and 13). We observe an increasing cloud expansion in the spanwise direction with the increase in wind speed. Moreover, at both wind speeds, the cloud retains a spherical-like shape at both t = 2 s and t = 3 s for all temperature and relative humidity values with increasing evaporation rate at low RH and high temperature. The above finding reinforces the recommendations that social distancing becomes important both in the streamwise (wind direction) and spanwise direction. Moreover, the present results could be used to set future prevention measurements in both indoor and outdoor environments to reduce airborne virus transmission by controlling the temperature, RH, and space ventilation rate.

**FIG. 14.**
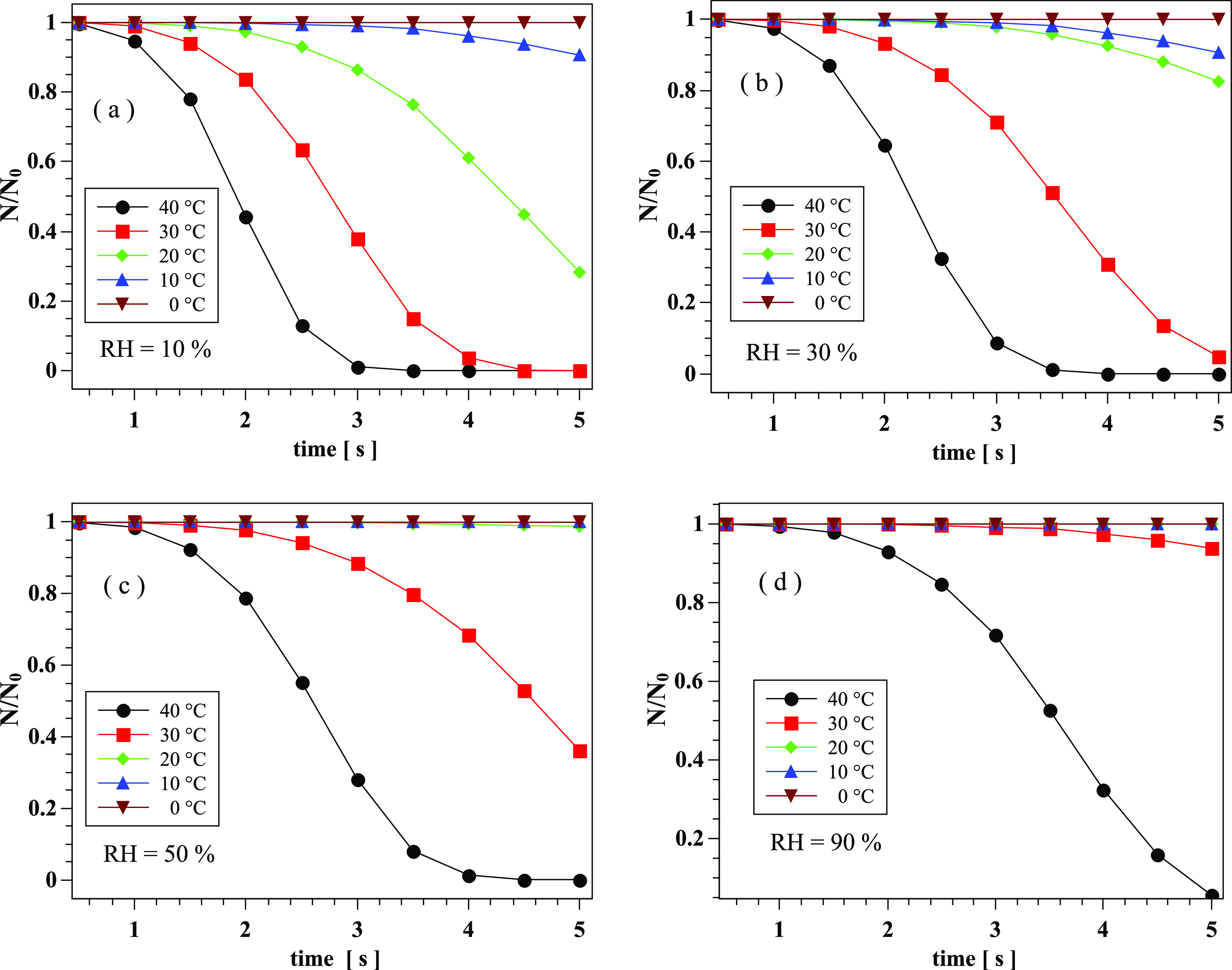
Weather effects on the transport and evaporation of airborne contaminated saliva droplets at *U*_*wind*_ = 4 km/h. Dimensionless droplet reduction with time at different temperatures and RH: (a): RH = 10%; (b): RH = 30%; (c): RH = 50%; and (d): RH = 90%.

The droplet number, *N*, compared to its initial value of *N*_0_(*t* = 0) = 1151 decreases at different rates due to evaporation (Fig. 14). At RH 90%, the droplet number reduces only when the temperature is at 40 °C, while it remains intact for temperatures up to almost 40 °C, with only a small reduction observed after 4.5 s at 30 °C. The droplet reduction becomes more significant at lower RH. For RH 50%, the reduction occurs for temperatures higher than 30 °C. For RH 30%, the temperature starts affecting the droplets after 3.5 s, with the droplet reduction still occurring at 30 °C and 40 °C. At the lowest RH of 10% considered here, we start seeing significant effects of temperature on droplet reduction at 20 °C and above.

## IV. CONCLUSIONS

We have developed new theoretical correlations for the Nusselt and Sherwood number and implemented their mathematical formulas in an Eulerian–Lagrangian multiphase CFD solver. These new correlations take into account the properties of the virus inside the saliva droplet, as well as the transient effects on heat and mass transfer.

We show that the steady-state theory leads to incorrect values of Nu and Sh numbers with the relative difference increasing as a function of the Reynolds number defined at the droplet scale [Eq. (1)]. The above means that the relative difference increases considerably with increasing wind speed or droplet diameter. As an example, we showed that at Re = 250, the relative difference could increase up to 600% at the instant of droplet-air impact with its time value being an increasing function of the droplet diameter. Therefore, the predictions of evaporation and virus concentration in saliva droplets are significantly underestimated if applying the widely used Ranz and Marshall correlation^11,12^ that is intended for heat and mass transfer of a single-material sphere at the steady-state. The latter correlation does not account for the effect of time (or thermal history kernel), and more importantly, the impact of multi-material (mixture) properties of CoV particles concentrated in saliva droplets.

In the second part of the study, using multiphase CFD models in conjunction with the new Nu and Sh correlations, we studied the effects of RH, temperature, and wind on the respiratory droplet transport and evaporation. Through several examples, we illustrated that high temperature and low relative humidity lead to high evaporation rates of saliva contaminated droplets, thus significantly reducing the virus viability. We quantified the evaporation rates as a function of the wind speed from three-dimensional CFD simulations. Additionally, we observed that the droplet cloud’s traveled distance and its concentration continue to be significant, even at high temperatures if the relative humidity is high too.

This study did not aim to link the evaporation rate change to disease transmission. This would be an impossible task because no one knows the viral load required for someone to be infected. The above may vary from one person to another and will depend on several factors such as age, gender, underlying medical conditions, and, possibly, genetic factors. Our work focuses on the potential risk of being infected—rather than disease transmission itself—from exposure to an airborne cloud of contaminated saliva droplets.

Our findings reinforce the importance of social distancing and the use of face masks to prevent full virus spread. The results reveal the importance of the weather conditions in the virus’s viability. They could guide the design of measures in both indoor and outdoor environments to reduce airborne virus transmission in private and public places.

## DATA AVAILABILITY

The data that support the findings of this study are available on request from the authors.

## References

[1] M. Richard, J. van den Brand, T. Bestebroer, P. Lexmond, D. de Meulder, R. Fouchier, A. Lowen, and S. Herfst, “Influenza a viruses are transmitted via the air from the nasal respiratory epithelium of ferrets,” Nat. Commun. 11, 766 (2020). 10.1038/s41467-020-14626-0 32034144 PMC7005743

[2] Z. Lei, Q. Yuhang, L.-F. Paolo, C. Yi, and Z. Yangying, “COVID-19: Effects of weather conditions on the propagation of respiratory droplets,” medRxiv (2020). 10.1101/2020.05.24.20111963

[3] R. Zhang, Y. Li, A. L. Zhang, Y. Wang, and M. J. Molina, “Identifying airborne transmission as the dominant route for the spread of COVID-19,” Proc. Natl. Acad. Sci. U. S. A. 117, 14857–14863 (2020). 10.1073/pnas.2009637117 32527856 PMC7334447

[4] C. B. Beggs, “The airborne transmission of infection in hospital buildings: Fact or fiction?,” Indoor Built Environ. 12, 9–18 (2003). 10.1177/1420326x03012001002

[5] B. Killingley and J. Nguyen-Van-Tam, “Routes of influenza transmission,” Influenza Other Respir. Viruses 7, 42–51 (2013). 10.1111/irv.12080 PMC5909391 24034483

[6] R. E. Davis, C. E. Rossier, and K. B. Enfield, “The impact of weather on influenza and pneumonia mortality in New York city, 1975–2002: A retrospective study,” PLoS One 7, e34091 (2012). 10.1371/journal.pone.0034091 22470518 PMC3314701

[7] K. M. Gustin, J. A. Belser, V. Veguilla, H. Zeng, J. M. Katz, T. M. Tumpey, and T. R. Maines, “Environmental conditions affect exhalation of h3n2 seasonal and variant influenza viruses and respiratory droplet transmission in ferrets,” PLoS One 10, e0125874 (2015). 10.1371/journal.pone.0125874 25969995 PMC4430532

[8] D. Zang, S. Tarafdar, Y. Yu. Tarasevich, M. Dutta Choudhury, and T. Dutta, “Evaporation of a droplet: From physics to applications,” Phys. Rep. 804, 1–56 (2019). 10.1016/j.physrep.2019.01.008

[9] S. S. Sazhin, “Advanced models of fuel droplet heating and evaporation,” Prog. Energy Combust. Sci. 32, 162–214 (2006). 10.1016/j.pecs.2005.11.001

[10] E. P. Vejerano and L. C. Marr, “Physico-chemical characteristics of evaporating respiratory fluid droplets,” J. R. Soc. Interface 15, 20170939 (2018). 10.1098/rsif.2017.0939 29491178 PMC5832737

[11] W. E. Ranz and W. R. Marshall, “Evaporation from drops, Part I,” Chem. Eng. Prog. 48, 141–146 (1952).

[12] W. E. Ranz and W. R. Marshall, “Evaporation from drops, Part II,” Chem. Eng. Prog. 48, 173–180 (1952).

[13] P. Mecenas, R. Bastos, A. Vallinoto, and D. Normando, “Effects of temperature and humidity on the spread of COVID-19: A systematic review.” medRxiv:20064923 (2020). 10.1371/journal.pone.0238339 PMC7500589 32946453

[14] Q. Li, X. Guan, P. Wu, X. Wang, L. Zhou *et al.*, “Early transmission dynamics in Wuhan, China, of novel coronavirus-infected pneumonia,” N. Engl. J. Med. 382(13), 1199–1207 (2020). 10.1056/NEJMoa2001316 31995857 PMC7121484

[15] C. I. Paules, H. D. Marston, and A. S. Fauci, “Coronavirus infections-more than just the common cold,” JAMA 323, 707–708 (2020). 10.1001/jama.2020.0757 31971553

[16] M. Moriyama, W. Hugentobler, and A. Iwasaki, “Seasonality of respiratory viral infections,” Annu. Rev. Virol. 7, 2.1–2.19 (2020). 10.1146/annurev-virology-012420-022445 32196426

[17] R. Bhardwaj and A. Agrawal, “Tailoring surface wettability to reduce chances of infection of COVID-19 by a respiratory droplet and to improve the effectiveness of personal protection equipment,” Phys. Fluids 32, 081702 (2020). 10.1063/5.0020249 PMC8728633 35002197

[18] R. Bhardwaj and A. Agrawal, “Likelihood of survival of coronavirus in a respiratory droplet deposited on a solid surface,” Phys. Fluids 32, 061704 (2020). 10.1063/5.0012009 PMC7295365 32574230

[19] K. H. Chan, J. S. Malik Peiris, S. Y. Lam, L. L. M. Poon, K. Y. Yuen, and W. H. Seto, “The effects of temperature and relative humidity on the viability of the SARS coronavirus.” Adv Virol. 2011, 1–7. 10.1155/2011/734690 PMC3265313 22312351

[20] K. K.-W. To, O. T.-Y. Tsang, C. C.-Y. Yip, K.-H. Chan, T.-C. Wu, J. M.-C. Chan, W.-S. Leung, T. S.-H. Chik, C. Y.-C. Choi, D. H. Kandamby, D. C. Lung, A. R. Tam, R. W.-S. Poon, A. Y.-F. Fung, I. F.-N. Hung, V. C.-C. Cheng, J. F.-W. Chan, and K.-Y. Yuen, “Consistent detection of 2019 novel coronavirus in saliva,” Clin. Infect. Dis. 71, 841–843 (2020). 10.1093/cid/ciaa149 32047895 PMC7108139

[21] R. Xu, B. Cui, X. Duan, P. Zhang, X. Zhou, and Q. Yuan, “Saliva: Potential diagnostic value and transmission of 2019-nCov,” Int. J. Oral Sci. 12, 1–6 (2020). 10.1038/s41368-020-0080-z 32300101 PMC7162686

[22] N. L’Helgouach, P. Champigneux, F. Santos-Schneider, L. Molina, J. Espeut, M. Alali, J. Baptiste, L. Cardeur, B. Dubuc, V. Foulongne, F. Galtier, A. Makinson, G. Marin, M.-C. Picot, A. Prieux-Lejeune, M. Quenot, F. J. Checa-Robles, N. Salvetat, D. Vetter, J. Reynes, and F. Molina, “EasyCOV : Lamp based rapid detection of SARS-COV-2 in saliva,” medRxiv:20117291 (2020).

[23] L. Azzi, G. Carcano, F. Gianfagna, P. Grossi, D. D. Gasperina, A. Genoni, M. Fasano, F. Sessa, L. Tettamanti, F. Carinci, V. Maurino, A. Rossi, A. Tagliabue, and A. Baj, “Salivais a reliable tool to detect SARS-CoV-2,” J. Infect. 81, e45–e50 (2020). 10.1016/j.jinf.2020.04.005 32298676 PMC7194805

[24] J. H. Azzolini, K. C. Winkler, and S. M. Kool, “Virus survival as a seasonal factor in influenza and poliomyelitis,” Nature 188, 430–431 (1960). 10.1038/188430a0 13713229

[25] J. Shaman, M. Kohn, and B. H. Singer, “Absolute humidity modulates influenza survival, transmission, and seasonality,” Proc. Natl. Acad. Sci. U. S. A. 106, 3243–3248 (2009). 10.1073/pnas.0806852106 19204283 PMC2651255

[26] J. Shaman, E. Goldstein, and M. Lipsitch, “Absolute humidity and pandemic versus epidemic influenza,” Am. J. Epidemiol. 173, 127–135 (2011). 10.1093/aje/kwq347 21081646 PMC3011950

[27] T. Myatt, M. Kaufman, J. Allen, D. MacIntosh, M. Fabian, and J. McDevitt, “Modeling the airborne survival of influenza virus in a residential setting: The impacts of home humidification,” Environ. Health 9, 55 (2010). 10.1186/1476-069X-9-55 20815876 PMC2940868

[28] W. Yang and L. Marr, “Mechanisms by which ambient humidity may affect viruses in aerosols,” Appl. Environ. Microbiol. 78, 6781–6788 (2012). 10.1128/AEM.01658-12 22820337 PMC3457514

[29] M. Sobsey and J. Meschke, “Virus survival in the environment with special attention to survival in sewage droplets and other environmental media of fecal or respiratory origin.” Res. Gate 1-71, 22855142 (2003).

[30] G. J. Harper, “Airborne micro-organisms: Survival tests with four viruses,” Epidemiol. Infect. 59(4), 479–486 (1961). 10.1017/s0022172400039176 PMC2134455 13904777

[31] S. J. Webb, R. Bather, and R. W. Hodges, “The effect of relative humidity and inositol on air-borne viruses,” Can. J. Microbiol. 9, 87–92 (1963). 10.1139/m63-009

[32] T. Dbouk and D. Drikakis, “On coughing and airborne droplet transmission to humans,” Phys. Fluids 32, 053310 (2020). 10.1063/5.0011960 PMC7239332 32574229

[33] I. B. Celik, U. Ghia, P. J. Roache, and C. J. Freitas, “Procedure for estimation and reporting of uncertainty due to discretization in CFD applications,” J. Fluids Eng. 130, 078001 (2008). 10.1115/1.2960953

[34] F. Moukalled, L. Mangani, and M. Darwish, The Finite Volume Method in Computational Fluid Dynamics: An Advanced Introduction with OpenFOAM and Matlab, 1st ed. (Springer Publishing Company, Incorporated, 2015).

[35] T. Miyoshi, M. Lönnfors, J. Peter Slotte, and S. Kato, “A detailed analysis of partial molecular volumes in DPPC/cholesterol binary bilayers,” Biochim. Biophys. Acta (BBA) 1838, 3069–3077 (2014). 10.1016/j.bbamem.2014.07.004 25151597

[36] C. Hidalgo, Physical Properties of Biological Membranes and Their Functional Implications (Plenum press, New York, London, 1988).

[37] S. Youssefian, N. Rahbar, C. R. Lambert, and S. Van Dessel., “Variation of thermal conductivity of DPPC lipid bilayer membranes around the phase transition temperature,” R. Soc. Interface 14, 20170127 (2017). 10.1098/rsif.2017.0127 28539484 PMC5454301

[38] W. Fuchs, “Z. d. sowj.-union6,” Physik 224 S (1934).

[39] W. F. Wells, “On air-borne infection: Study II. Droplets and droplet nuclei,” Am. J. Hyg. 20, 611–618 (1934). 10.1093/oxfordjournals.aje.a118097

[40] N. Frossling, “Uber die verdunstung fallender tropfen (the evaporation of falling drops),” Gerlands Beitrage Geophys. 52 (1938).

[41] A. Acrivos and T. D. Taylor, “Heat and mass transfer from single spheres in Stokes flow,” Phys. Fluids 5, 387–394 (1962). 10.1063/1.1706630

[42] H. Brenner, “Forced convection heat and mass transfer at small Péclet numbers from a particle of arbitrary shape,” Chem. Eng. Sci. 18, 109–122 (1963). 10.1016/0009-2509(63)80020-2

[43] P. D. Richardson, WADD 59-1, 1968.

[44] S. Whitaker, “Forced convection heat transfer correlations for flow in pipes, past flat plates, single cylinders, single spheres, and for flow in packed beds and tube bundles,” AIChE J. 18, 361–371 (1972). 10.1002/aic.690180219

[45] H. Kramers, “Heat transfer from spheres to flowing media,” Physica 12, 61–80 (1946). 10.1016/S0031-8914(46)80024-7

[46] C. Vliet and J. C. Leppert, “Closure to “discussions of ‘forced convection heat transfer from an isothermal sphere to water’” (1961, ASME J. Heat Transfer, 83, pp. 170–175),” J. Heat Trarnsfer 83, 170–175 (1961). 10.1115/1.3680509

[47] Z.-G. Feng and E. E. Michaelides, “Unsteady heat transfer from a sphere at small Péclet numbers,” J. Fluid Eng. 118, 96–102 (1996). 10.1115/1.2817522

[48] Z.-G. Feng and E. E. Michaelides, “Transient heat transfer from a particle with arbitrary shape and motion,” J. Heat Transfer 120, 674–681 (1998). 10.1115/1.2824336

[49] Z.-G. Feng and E. E. Michaelides, “A numerical study on the transient heat transfer from a sphere at high Reynolds and Péclet numbers,” Int. J. Heat Mass Transfer 43, 219–229 (2000). 10.1016/s0017-9310(99)00133-7

[50] Z.-G. Feng and E. E. Michaelides, “Heat and mass transfer coefficients of viscous spheres,” Int. J. Heat Mass Transfer 44, 4445–4454 (2001). 10.1016/s0017-9310(01)00090-4

[51] Z. Duan, B. He, and Y. Duan, “Sphere drag and heat transfer,” Sci. Rep. 5, 1–7 (2015). 10.1038/srep12304 PMC4648403 26189698

[52] P. Yearling and R. Gould, Convective Heat and Mass Transfer from Single Evaporating Water, Methanol and Ethanol Droplets (American Society of Mechanical Engineers, New York, NY, USA, 1995).

[53] E. Pfender, “Heat and momentum transfer to particles in thermal plasma flows,” Pure Appl. Chem. 57(9), 1179–1195 (1985). 10.1351/pac198557091179

[54] T. H. Chilton and A. Colburn, “Mass transfer (absorption) coefficients prediction from data on heat transfer and fluid friction,” Ind. Eng. Chem. 26, 1183–1187 (1934). 10.1021/ie50299a012

[55] E. Pohlhausen, “Der wärmeaustausch zwischen festen körpern und flüssigkeiten mit kleiner reibung und kleiner wärmeleitung,” ZAMM-J. Angew. Math Mech. 1, 115–121 (1921). 10.1002/zamm.19210010205

[56] T. Dbouk and D. Drikakis, “On respiratory droplets and face masks,” Phys. Fluids 32, 063303 (2020). 10.1063/5.0015044 PMC7301882 32574231

[57] V. Vinoj, N. Gopinath, K. Landu, B. Behera, and B. Mishra, “The COVID-19 spread in India and its dependence on temperature and relative humidity,” arXiv:2020070082 (2020).

